# Inkjet-based surface structuring: amplifying sweetness perception through additive manufacturing in foods

**DOI:** 10.1038/s41538-023-00218-x

**Published:** 2023-08-18

**Authors:** Johannes Burkard, Lucas Kohler, Tanja Berger, Mitsuko Logean, Kim Mishra, Erich J. Windhab, Christoph Denkel

**Affiliations:** 1 Insitute of Food, Nutrition and Health, ETH Zürich, Zürich, Switzerland; 2 School of Agricultural, Forest and Food Sciences, Food Science and Management, Bern University of Applied Sciences, Zollikofen, Switzerland

**Keywords:** Health care, Engineering

## Abstract

Additive manufacturing (AM) is creating new possibilities for innovative tailoring of food properties through multiscale structuring. This research investigated a high-speed inkjet-based technique aimed to modify sweetness perception by creating dot patterns on chocolate surfaces. The dots were formulated from cocoa butter with emulsified water droplets containing the sweetener thaumatin. The number and surface arrangement of dots, which ranged from uniformly distributed patterns to concentrated configurations at the sample’s center and periphery, were varied while maintaining a constant total amount of thaumatin per sample. A sensory panel evaluated sweetness perception at three consumption time points, reporting a significant increase when thaumatin was concentrated on the surface. Specifically, an amplification of sweetness perception by up to 300% was observed, irrespective of dot pattern or consumption time, when compared to samples where thaumatin was uniformly distributed throughout the bulk. However, when thaumatin was concentrated solely at the sample center, maximum sweetness perception decreased by 24%. Conclusively, both the proximity of thaumatin to taste receptors and its spatial distribution, governed by different dot arrangements, significantly influenced taste responsiveness. These findings present a more effective technique to substantially enhance sweetness perception compared to traditional manufacturing techniques. This method concurrently allows for sensorial and visual customization of products. The implications of this study are far-reaching, opening avenues for industrially relevant AM applications, and innovative approaches to study taste formation and perception during oral processing of foods.

## Introduction

Obesity, a widespread issue closely linked to dietary practices^1,2^, has prompted substantial efforts to reduce sugar and fat intake while maintaining desirable sensory perception^3–9^. To lower calorie content, common strategies involve substituting sugar with non-nutritive sweeteners, often combined with fibers or sugar alcohols to replace the bulk of sucrose^10–12^. However, these modifications may adversely impact flavor^13,14^. Another strategy involves enhancing perceived sweetness through a multisensory approach^15,16^, such as adding strawberry odor to whipped cream to heighten sweet-ness perception^11^.

Researchers have recently explored the potential of modifying taste perception by altering food structure^12,17,18^. For instance, it has been shown that saltiness perception can differ between emulsions and aqueous solutions^19^, or that sweetness perception can be increased in gelled semi-solid systems with uneven sugar distribution^18^. Furthermore, it has been possible to reduce sucrose concentration by up to 20% in agar/gelatine composite gels without impacting sweetness perception^17^.

Additive manufacturing (AM) offers the capability to realize more complex structural designs compared to the limitations of manual layering. For example, Kistler et al.^20^ created chocolate-hydrocolloid composites with varying macroscopic sweetness gradients using fused deposition modeling (FDM), reporting a 30% increase in sweetness perception for a cube-in-cube prototype. Inkjet printing, which offers high precision printing^21^, has been used to deposit chocolate layers of varying thicknesses onto rice waffles, enhancing sweetness, creaminess, and fullness perceptions^22^.

This study aimed to understand how the spatial distribution of thaumatin, a model sweetener, affects sweetness perception. A novel inkjet-based technique was used to create distinct dot patterns on chocolate, controlling the spatial distribution of thaumatin. Thaumatin, solubilized in water, was emulsified and incorporated into a semi-crystalline cocoa butter matrix, resulting in printable masses referred to as inks. By managing the ink’s melting and flow properties, and measuring dot shape and size, desired dot texture and print quality were ensured. Seven samples were created with varying dot patterns and thaumatin concentrations (independent variables), while maintaining a constant total thaumatin amount. These samples were categorized as micro- or mesoscopic, terms indicating the dot arrangement, and were compared with a reference sample with evenly dispersed thaumatin in the chocolate bulk. Fourteen to seventeen panelists, who received extensive training, evaluated perceived sweetness intensity at three consumption time points using a sensory scale. These evaluations served as the dependent variables for the study. Based on these sensory evaluations, a mechanism was proposed to explain the observed outcomes to more accurately develop novel AM food designs.

## Results

### Instrumental comparison of the printable ink masses

**Fig. 1:**
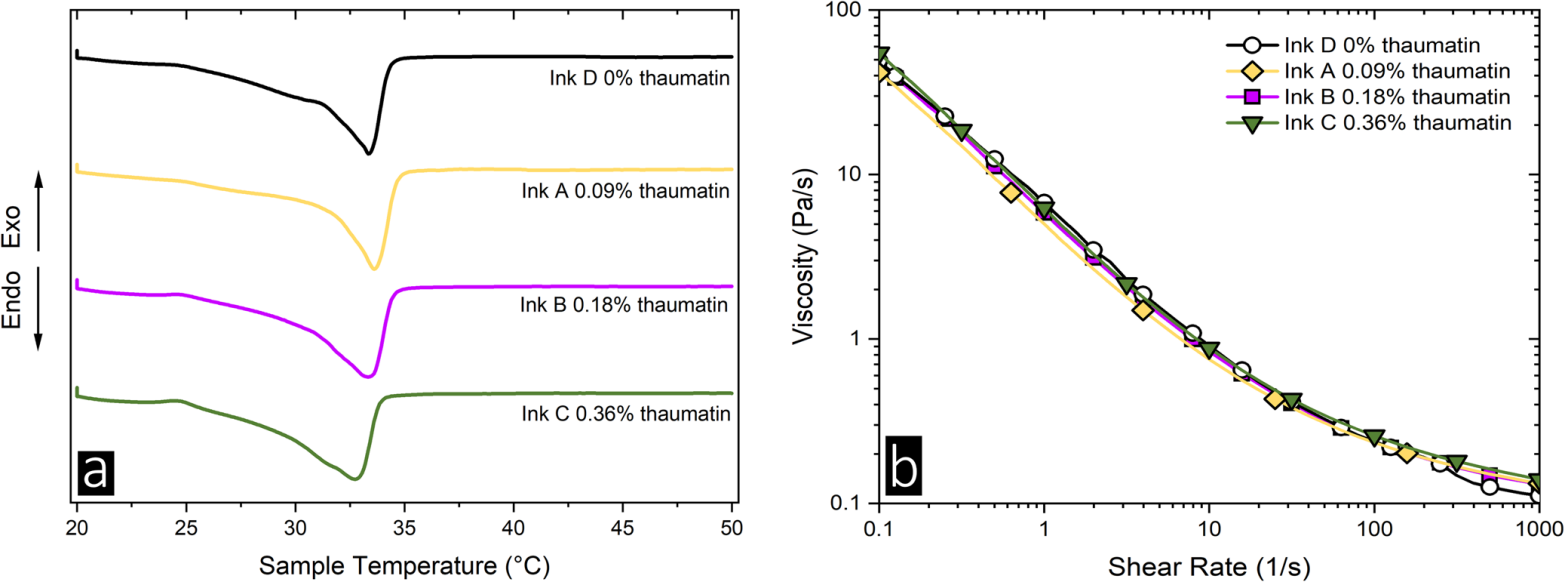
Calorimetry and rheology analysis of inks a to d. **a** Differential Scanning Calorimetry of inks a, b, c, and d over a temperature range from 20 to 50 °C. **b** Rotational rheology of the inks between 0.1 and 1000 s^−1^. Viscosity curves are depicted as a function of shear rate. Values are presented as means. Standard deviations (sd) were calculated but are not shown in the figure for clarity of presentation.

Calorimetry and rheology analysis on inks A to D (Table 1) were performed to rule out any textural differences that could influence perceived sensory sweetness, as described in previous studies^23^. Regardless of thaumatin concentration in inks A to D, the melting range was found between 33.3 °C and 33.7 °C, indicating that melting properties and crystal structures (*β*_V_) were not affected by thaumatin concentration^24^. Thereby, comparable melting properties for all ink masses during consumption can be anticipated. As detailed in Fig. 1b, all inks displayed a common shear-thinning behavior, consistent with the literature on cocoa butter crystal-melt suspensions^25,26^. No hysteresis effect was observed in the up - and downward shear rate ramp, in line with Mishra et al.^27^.

**Table 1:** Composition of the inks A to D (% w/w).

Material	Surface Printable Inks (% w/w)			
	A	B	C	D
**Seeded Cocoa Butter**	**0.9**	**0.9**	**0.9**	**0.9**
**Emulsion**	**0.1**	**0.1**	**0.1**	**0.1**
*containing (% w/w):*				
Thaumatin	0.9	1.8	3.6	0
Water	44.1	43.2	41.4	45
PGPR	3	3	3	3
CCT-Oil	52	52	52	52

**Fig. 2:**
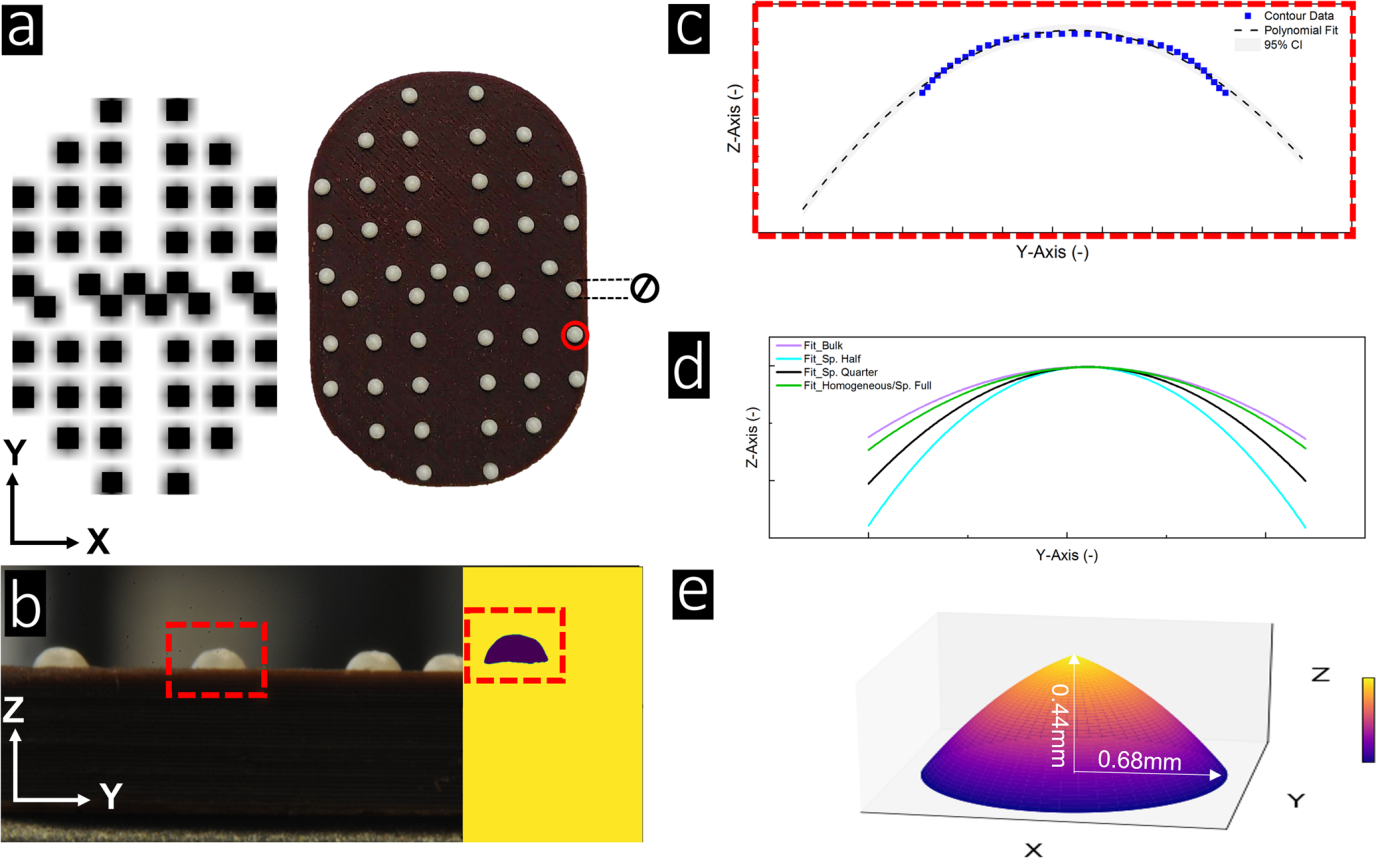
Dot contours - from bitmap to printed patterns. **a** Top view of the printed dot pattern for *Sp. Quarter*, created with ink of composition C, is displayed alongside the original bitmap pattern design. Additional dot patterns, featuring varying ink thaumatin concentrations, can be found in Supplementary Fig. 1. Dot diameter was averaged from top view images for surface reconstruction. For illustrative purposes, a side view of the sample and a specific dot (both highlighted in red) is presented in **b**. This image underwent binarization and image processing. The contours of the selected dot surface (highlighted by the red frame) were fitted with a second-degree polynomial function, with the 95 % confidence interval visualized as a gray hue, as depicted in **c**. The average contour fittings from all samples with different patterns are visualized in **d**. By utilizing the extracted radii from (a) and the polynomial fit from **d**, the dot surface was approximated as a paraboloid, as demonstrated in **e**. For a comprehensive workflow, please refer to Supplementary Note 1.

While careful regulation of ink flow and melting was in place, there were variations in dot radius - ranging from 0.68 mm in those printed with ink B to 0.8 mm in those printed with ink D/A (see Fig. 2e and Supplementary Fig. 1). Dot height also varied from 0.32 mm with ink D to 0.54 mm with ink B. These discrepancies are more likely attributable to minor fluctuations in product temperature during the printing process rather than variances brought on by the concentration of thaumatin. However, despite these slight variations, overall surface area of the dots remained consistent, falling within the range of 2.15 to 2.3 mm^2^. This consistency, confirmed by sensory panel evaluations, suggests that any potential perceptual differences during licking due to changes in ink melting or flow properties, or dot shape, would likely be negligible or insignificant.

### Sensory evaluation

#### Sample modifications on the microscale level

**Fig. 3:**
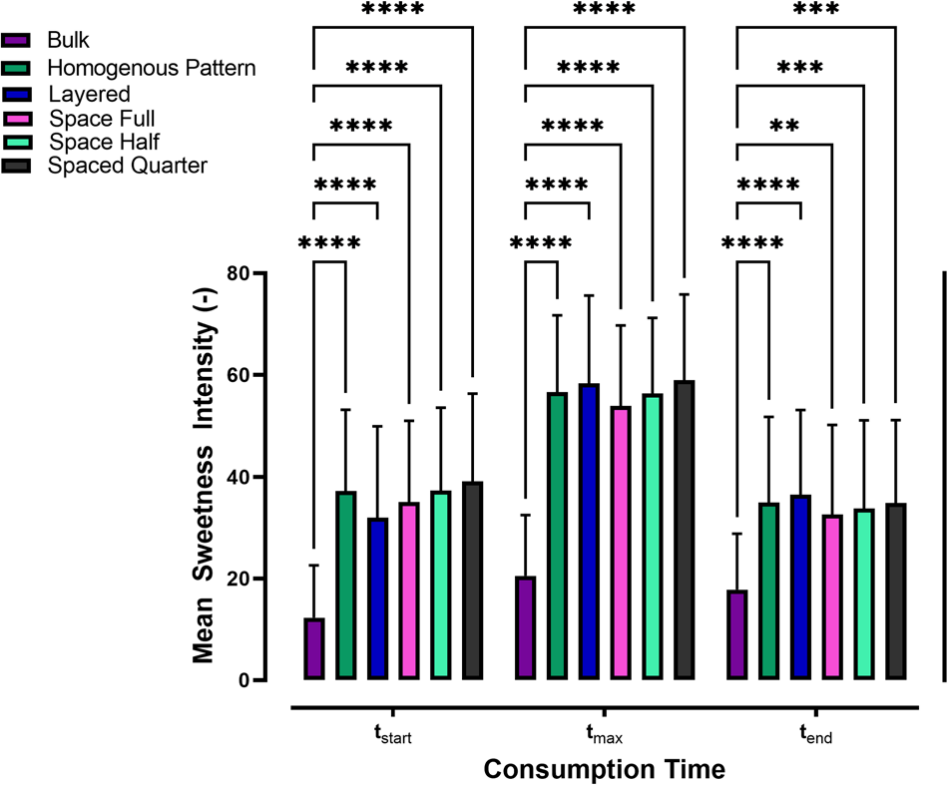
Sweetness intensity variations at the microscale level. Mean sweetness intensity ratings of all samples with patterns varying on a microscale level, summarized for both test sessions and all participants. Each bar also includes error bars representing the s.d. of the mean. Signifi- cance between samples at each time point was evaluated using a two-way ANOVA and a post-hoc Tukey test. Only significant differences are shown in the plot. The number of asterisks (*) represents different levels of significance (** *<* 0.1, *** *<* 0.01, **** *<* 0.001).

Figure 3 shows perceived sweetness intensity at three time points t_start_, t_max_, and t_end_. The reference sample, referred to as *Bulk*, was formulated to mimic a standard chocolate composition. However, in this sample, sucrose was substituted with a combination of inulin/polydextrose and thaumatin. This substitution aimed to replicate both the bulking properties and sweetness intensity that would typically be provided by sucrose. Comparing the dotted samples to the reference sample, it was observed that sweetness intensity was notably higher in the dotted samples. At t_start_ and t_max_, the dotted samples exhibited sweetness amplification of up to 300%, while at t_end_, the amplification was up to 200%. Yet, there were no significant differences among the micro-structured samples. These findings underscore the potential of surface-positioned tastants, in this case, thaumatin, to considerably enhance and extend its perception.

The enhanced sweetness perception observed in the dotted samples can be attributed to the closer proximity of thaumatin to taste receptors and its increased mobility within the aqueous phase of the fat-continuous ink^28,29^. In contrast, when thaumatin was dispersed in the bulk matrix, its release rate into the sample-saliva interface during oral processing was reduced. This reduction can be attributed to both the lower concentration of thaumatin and the increased viscosity of the chocolate bulk - both of which are recognized factors that influence taste perception^30,31^. As consumption progressed, sample movement and interfacial renewal gradually cleared thaumatin from taste receptors, resulting in the observed decrease in sweetness intensities for all samples towards t_end_.

#### Sample modifications on the mesoscale level

**Fig. 4:**
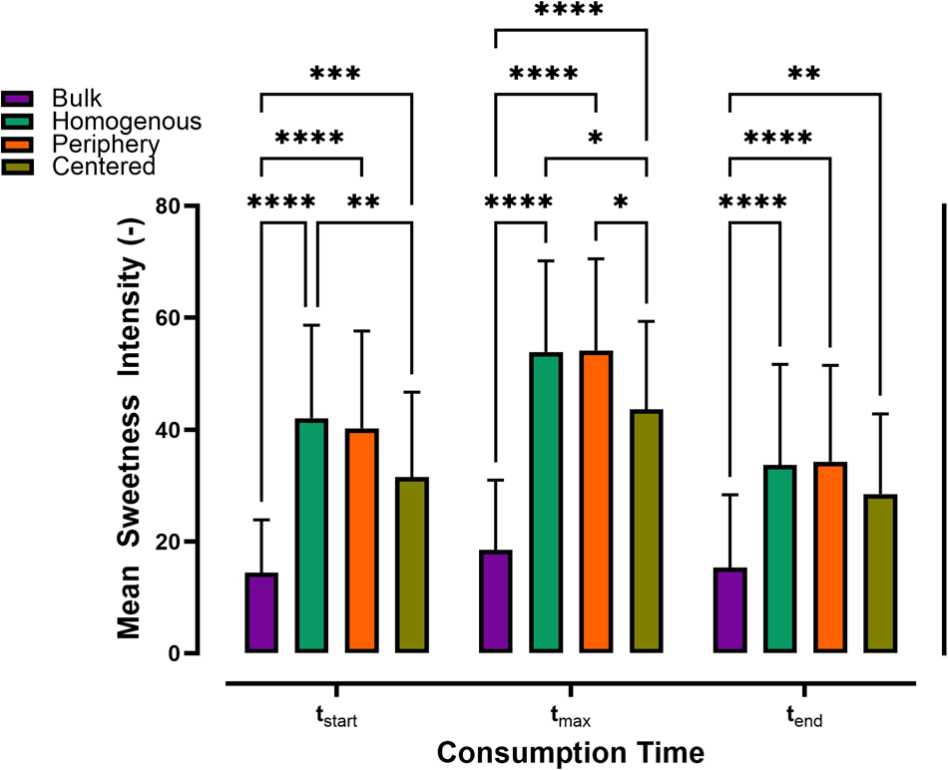
Sweetness intensity variations at the mesoscale level. Mean sweetness intensity ratings of the samples with different dot patterns on mesoscale level, summarized for both test sessions and all participants. Each bar also includes error bars representing the s.d. of the mean. Signifi- cance between samples at each time point was evaluated using a two-way ANOVA and a post-hoc Tukey test. Only significant differences are shown in the plot. The number of asterisks (*) represents different levels of signifi- cance (* *<* 0.5, ** *<* 0.1, *** *<* 0.01, **** *<* 0.001).

In this experiment, thaumatin-loaded dots were positioned either at the center or the periphery of the sample surfaces, ensuring equal overall thaumatin content and the total number of dots on each surface. Figure 4 shows that all samples with dot patterns were perceived as sweeter than the reference *Bulk*. Among these, sample *Centered* was consistently perceived as the least sweet during consumption, with a notable 24% lower sweetness intensity compared to the sample *Periphery* at t_max_. These results indicate that the mesoscale level arrangement of thaumatin persisted during the tasting and led to distinct sweetness perceptions among the samples *Centered* and *Periphery*.

**Fig. 5:**
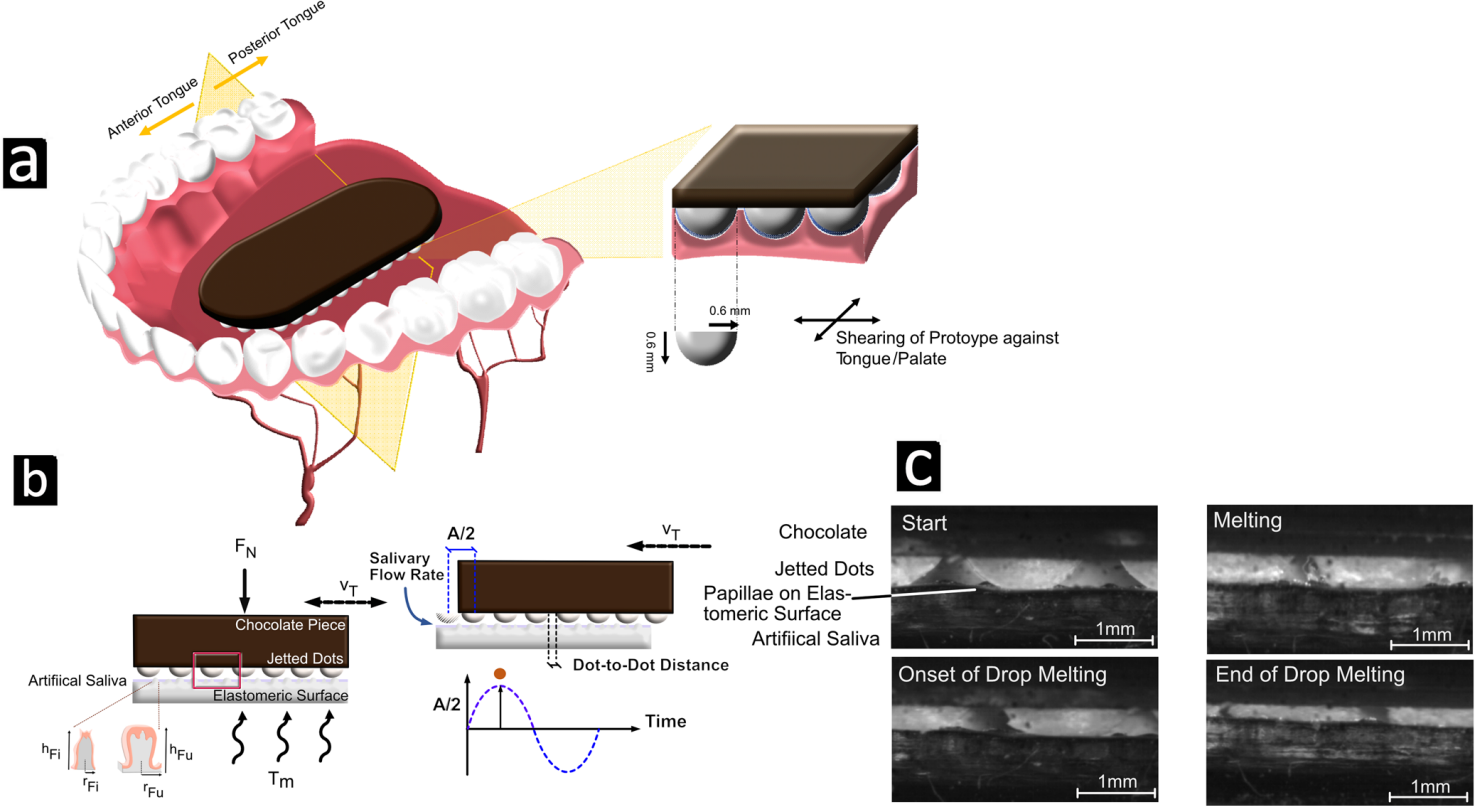
Design of tongue-inspired biomimetic surface. **a** Diagram of the mouth arch with a chocolate prototype, where the yellow plane approximately indicates the separation between anterior and posterior tongue regions with their distinct afferent nerves, the chorda tympani nerve (cranial nerve VII) and the glossopharyngeal nerve (cranial nerve IX). On the right, the boundary region between the tongue and chocolate/dots is shown at a greater magnification. The elastic tongue completely coats the semi-spherical surfaces of the dots, and the sample is sheared against the tongue/palate during consumption. **b** Diagram of the in-vitro simulation of drop melting, using a tongue-emulating elastomeric surface. The elastomer contained two types of human papillae (i.e., filiform and fungiform) that were produced by additive manufacturing. Sample consumption was emulated by oscillating the chocolate sample at a velocity v_T_ of 10 mm/s, at a normal force F_N_ of 1 N, and over an amplitude of sample-to-tongue A/2 of 5 mm. Human tongue movement and in-mouth normal force have been estimated to be in the range applied in our experimental setup^44,45^. **c** Dot melting is illustrated in four exemplary photos: At the beginning, the dot shape was completely intact and was sheared against the papillae while the tongue was slowly deforming under the normal force applied. In the later stages, the dot matrix was incrementally distributed close to the initial dot-to-tongue position, at maximum distances restricted by the amplitude between the sample and the tongue surface. A time grid of more selected images can be viewed in Supplementary Fig. 3.

In summary, taste intensity was influenced by thaumatin concentration and initial sample surface area. Yet, the current findings emphasize the importance of spatial gradients of thaumatin across the tongue surface in relation to perceived sweetness intensity. Previous studies have reported lower taste detection thresholds and greater taste sensations at the tip of the tongue than at its back^32,33^. It is plausible that samples like *Homogeneous* and *Periphery*, which simultaneously stimulated the tip and the back of the tongue, resulted in stronger perceptions due to the activation of two separate gustatory nerves associated with these regions, as illustrated in Fig. 5a.

#### Impact of the initial dot pattern on sweetness perception

During the initial phase of consumption, licking encouraged the shearing of the dotted samples against the tongue. In this process, melting dots released thaumatin into the saliva, facilitating its diffusion to taste receptor cells—an essential step in evoking sweetness perception^34^. This transfer was likely enhanced by the deformability of the tongue^35^, as consistent coverage of each dot by the tongue ensured uniform heat transfer and dot melting rates across all samples (Fig. 5a). Even though sample *Spaced Full* contained twice the number of dots and consequently also twice the amount of oil as sample *Spaced Half*, no perceptual differences could be observed. This suggests a quick release of thaumatin across all samples, possibly facilitated by the presence of PGPR^36,37^.

To better understand the governing mechanisms at the early stages of consumption, we utilized a tongue-inspired biomimetic surface designed to emulate the human tongue’s action during dot melting (Fig. 5b, c). As the dots began to melt, the liquefied mass spread across the tongue due to its oscillatory motion during consumption (see Supplementary Fig. 3B). This was evidenced by similar sweetness intensity ratings for samples *Homogeneous* and *Sp. Quarter*. Findings suggest that the oscillation amplitude of the tongue exceeded the dot-to-dot distance in *Sp. Quarter*, which at approximately 2.5 mm was the largest among all microscale dot patterns. Consequently, the resolution of dot patterns appeared to be too refined to the oral processing conditions that promoted a rapid spread within the oscillation range. However, when dot distances increased as observed in samples *Periphery* and *Centered* (clustered dot-to-dot distance exceeding *>* 8 mm), sweetness perception was altered. Given that the average distance between fungiform papillae—these are the most prominent papillae at the tongue tip containing taste buds^38^—falls within the reported range of 1 to 4 mm^39,40^, which aligns with the maximum dot distance of the micro-structured samples. This anatomical feature, coupled with horizontal shearing, could enable a more uniform taste experience for micro-structured samples. However, when the spaces between areas rich in thaumatin were larger, as seen in the sample *Periphery*, it is believed that multiple taste regions were triggered, resulting in altered sweetness perceptions.

**Fig. 6:**
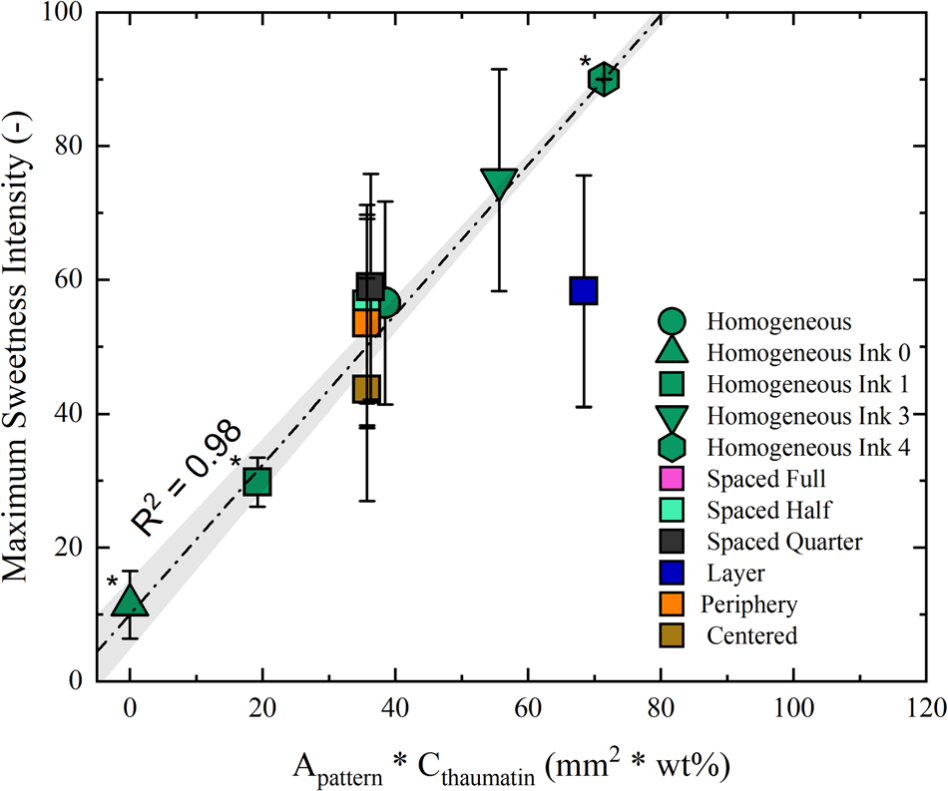
Relationship between stimulus size and sweetness perception. Maximum sweetness intensity as a function of initial stimulus size *A*_*pattern*_ * C_*thaumatin*_. Every data point represents the averaged maximum sweetness intensity of a sample, with error bars representing the s.d. The asterisk next to some labels appears for samples that were tested during sensory training only. The line represents the linear fit of the data array and the gray-shaded area shows the 95% confidence interval.

Our data suggest that the initial perception of sweetness was primarily driven by the tongue-dots contact area, which promoted thaumatin release during oral processing. This release seems to rely on both the initial surface area accessible for heat transfer (termed *A*_*pattern*_) and thaumatin concentration in the dots (*C*_*thaumatin*_), both of which are referred to as *stimulus size*. These factors appeared to influence thaumatin transfer equally. To substantiate this relationship, samples from training sessions were included that encompassed stimulus sizes both larger and smaller than those described in sub-chapters *Sample Modifications on the Micro-/Mesoscale Level*. The linear relationship shown in Fig. 6 (*R*^2^ = 0.98) reinforces this notion, indicating that both concentration of thaumatin in the dots and the area of stimulus (*A*_pattern_*C_*thaumatin*_) have a proportional impact on perceived intensity. In the manually assembled sample *Layer*, it was observed that without modifying the surface area through jetting, perceived sweetness intensity for its stimulus size was notably reduced. This relationship implies that when thaumatin-loaded dots are closely spaced (microscale level), sweetness perception is translated linearly from stimulus size, at least for the dotted samples.

Potential confounding factors should be acknowledged, such as the circumstance of at-home sensory tasting (due to COVID restrictions), visual differences between samples, and the subtle influence of hedonics on taste perception. Despite the limitations of our tasting protocol, most studies examining sensory attributes and perception often simplify the food matrix to link structural changes to perceptual observations more easily, such as in hydrocolloid systems. Future modeling should account for the many complex processes occurring during oral processing, like sample elongation or lubrication effects. Our combination of in-vitro oral processing emulation and rapid ink jetting setup may provide insights into the relationship between food matrix and tastant release.

## Discussion

This study employed inkjet printing to create patterns of thaumatin-filled dots on chocolate samples, while keeping total amount of sweetener constant. This method significantly enhanced perceived sweetness by up to 300% during consumption, offering a considerable advantage over traditional manufacturing where sweeteners or sugars are uniformly spread throughout the sample. Crucially, proximity to taste buds, in conjunction with physical processes and cognitive mechanisms linked to taste perception, played a pivotal role in enhancing sweetness sensation. Minor differences in sweetness intensity among micro-structured samples suggest that initial dot patterns may have blurred due to melting and movement, leading to a homogeneous thaumatin concentration across the tongue.

Perception of sweetness was primarily governed by the initial contact area between the tongue and dots, which affected heat transfer, and concentration of thaumatin, which modulated its release rate. The identified linear correlation between these parameters and sweetness intensity implies that varying patterns with different distances between thaumatin-filled dots did not influence sweetness; instead, sweetness intensity appears to average across all taste receptors. We also examined the effect of concentrating thaumatin-filled dots in the center or periphery of the sample. It was found that overall sweetness perception decreased when thaumatin was absent from the tip and back of the tongue, indicating local taste sensitivity of the tongue. We propose that activating taste receptors spaced sufficiently apart can influence sweetness perception in such cases.

This research offers a method for food manufacturers to enhance taste perception by creating local gradients, in parallel making visually attractive surface designs possible. Compared to traditional layering, our surface structuring technique allows for more flexible modification of perception by adjusting the pattern and dot density. The use of inkjet printing to selectively coat complex, non-flat surfaces offers a precise and adaptable tool for sensory modification. This opens up new avenues for sensory research, enabling more detailed investigations into locally-resolved sensory perception at multiple scales. Our method of positioning tastants could provide new strategies for understanding the spatial and time-resolved formation of sensory responses, bridging the gap from the food matrix to brain signal processing. This investigation underlines the importance of spatially distributed stimuli in taste perception and sets the stage for future research in this intriguing field.

## Methods

Chocolate samples were produced using cacao mass “Rondo” and cocoa butter (Max Felchlin AG, Ibach, Switzerland). Soy lecithin type EM04B was purchased from Max Felchlin AG (Ibach, Switzerland). Inulin type Orafti®HSI was kindly donated by BENEO-Orafti N.V. (Belgium). Polydextrose type Litesse® Two IP powder was purchased from Danisco AG (Rotterdam, Netherlands). A mix of polydextrose (26.33 %w/w) and inulin (11.73 %w/w) was introduced to replace sucrose technofunctionally as a bulking agent. These low-digestible carbohydrates were mixed with melted cacao mass (55.7 %w/w) and subsequently pre-mixed in a heated mixing device (Kenwood Major Titantium KMT056, Kenwood Swiss AG, Switzerland) for 30 minutes. In a second step, the premix was transferred to a melanger (ECGC-12 SLTA, Cocoatown LLC, USA) operating at 135 rpm. The melanger was placed in a temperature-controlled chamber, maintaining a sample temperature of 53 °C ± 3 °C. After 90 minutes, melted cocoa butter (4.85 %w/w) and soy lecithin (0.39 %w/w) were added to the conched mass, which was treated for another 270 minutes.

The conched chocolate mass was quenched to 34 °C in a heating chamber (Julabo TW8, Faust Laborbedarf, Switzerland) set at 34 °C. In total 1%w/w of seeded cocoa butter, produced in a SeedMaster Cryst (Bu¨hler AG, Uzwil, Switzerland), was added to the chocolate mass and manually mixed in for three minutes. A detailed workflow of the SeedMaster Cryst can be found in ref. ^26^. The final chocolate samples (2.34 g ± 0.07 g) were prepared by casting the tempered chocolate into silicone molds (Siliconen Culinair®). The geometric design of the molds is shown in Fig. 8, with a height of 3 mm, a total length of 36 mm, a width of 24 mm and tilted edges to facilitate consumption. After crystallization, the chocolate plates were detached from the silicone mold and stored at 4 °C.

### Preparation of printable ink masses

Thaumatin was purchased from Penta Manufacturing (New Jersey, USA). The printable ink was prepared by reconstituting thaumatin in Evian water (Evian, Cachat, France) at different concentrations, corresponding to ink formulations with variable sweetness potencies, detailed in Table 1. The solution was stirred with a magnetic stirrer at 600 rpm for one hour (Ika Yellowline MST basic c, Staufen im Breisgau, Germany). Caprylic capric triglycerides (CCT) (Mibelle Group, Buchs, Switzerland, Lot 10339) and polyglycerol poly-crinoleate (PGPR) (Danisco, Rotterdam, Netherlands) were added to the aqueous solution and were mixed with a Polytron mixer at 3000 rpm for two minutes (Kinematica GmbH, Kriens Luzern, Switzerland). The premix was transferred to a Microfluidizer processor (M-110EH Microfluidics, Newton MA, USA), where the mix was emulsified for six cycles at 1000 bar to form a W/O emulsion. Seeded cocoa butter was manufactured in a SeedMaster (SeedMas- ter cryst, Bu¨hler AG, Switzerland) for 150 min (see details in^41^) and was added to the emulsion at a weight ratio of 9:1. The ink (short for print- able ink mass) was carefully stirred for three minutes and either transferred to a preheated cartridge at 33.0 ± 0.1 °C or stored for rheology and calorimetric analysis, as detailed in the following subchapters.

### Rheology

Flow behavior of the printable inks A to D (see Table 1) was measured with a Rheometer MCR 100 (Physica, Anton Paar, Austria), applying a steady shear in a cylindrical Couette geometry (CC27, Anton Paar, Graz, Austria). Sample temperature was equilibrated at 33 °C for five minutes, before pre-shearing at 1000 s^−1^ for another 10 min. A logarithmic shear rate ramp between 0.1 s^−1^ and 1000 s^−1^ with ten data points per decade was performed, recording upward and downward ramps to study hysteresis effects. All measurements were performed in triplicates (*N* = 3).

### Differential scanning calorimetry

The melting behavior of the printable inks was investigated with a differential scanning calorimeter (DSC 3+/500, Mettler Toledo GmbH, Switzerland). An indium sample and a water sample were used for calibration. For measurement, 5 mg ± 0.5 mg of the sample was weighed into a 40 µl aluminum crucible (Mettler Toledo GmbH, Switzerland). The sample was equilibrated at 20 °C for 20 minutes and heated to 40 °C at 4 K/min. Based on the heat flow rate, different curve characteristics such as onset, offset, and peak melting temperatures were extracted and analyzed in OriginPro (2021). Each sample was measured in triplicates (*N* = 3).

### Surface inkjet printing

**Fig. 7:**
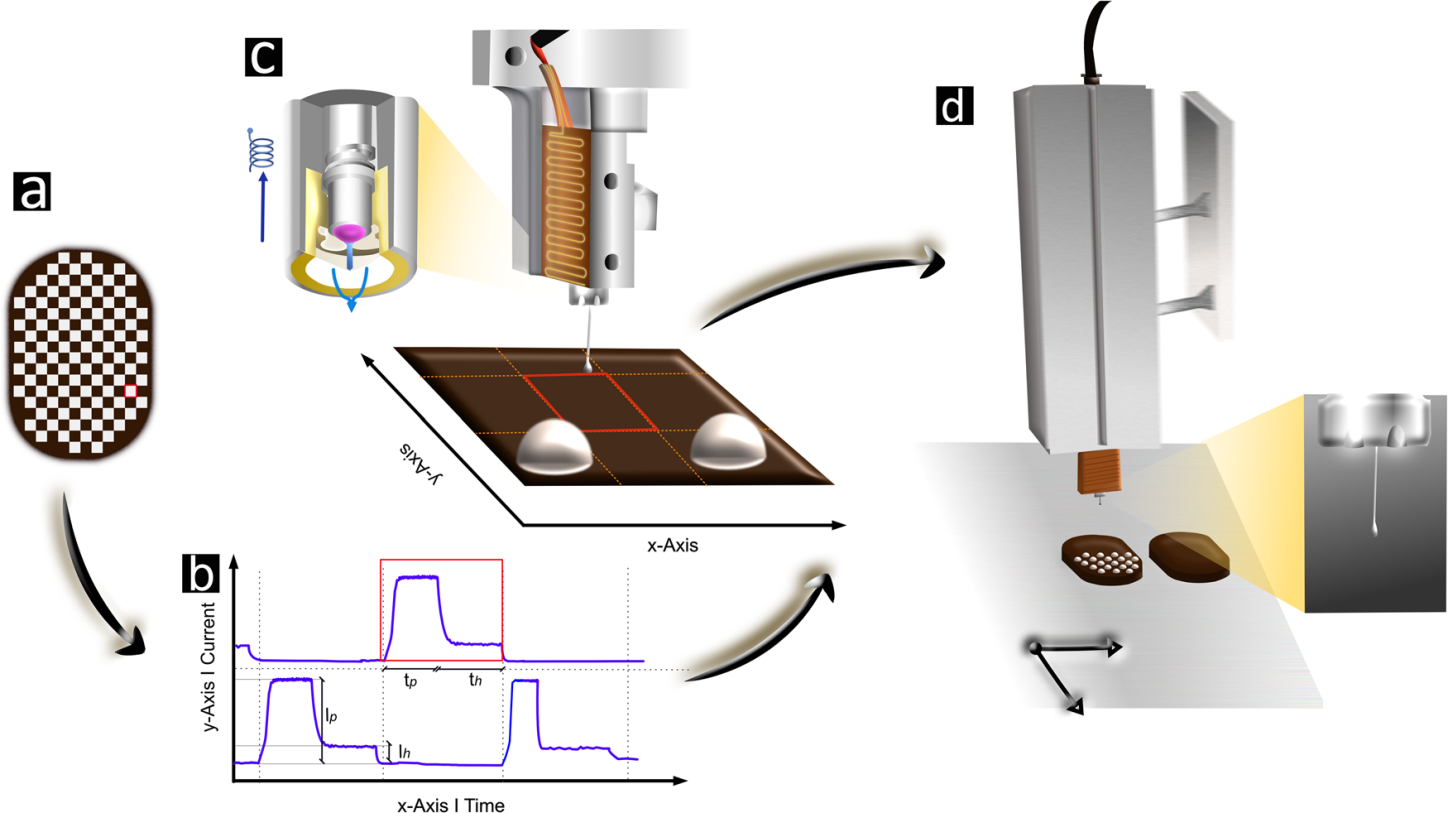
Schematic representation of the inkjet printing work-flow. Schematic representation of the inkjet printing workflow. **a** White pixels in the bitmap represent dots to be printed and black pixels, empty spaces. The complete workflow from translating the pattern to inkjet printing is illustrated with reference to the red-framed pixel. **b** The bitmap was rasterized and configured to valve and print commands, where valve opening was defined by the “peak-and-hold” procedure: A peak current *I*_*p*_ was initiated for a time *t*_*p*_, when the printer scanned a printable region. Afterwards, the valve visualized in **c** was kept open for a holding time *t*_*h*_ at a current *I*_*h*_ to shape drop volume. (c) The drop was formed once the valve ball was pulled back electromagnetically to let the medium emerge over a defined raster region. **d** The print head was installed on a cartridge, where the medium was kept at a constant temperature and conveyed pneumatically. Drop detachment was monitored with a dropwatching unit.

The printing device, built by the Institute for Print Technology (IDT, Bern University of Applied Sciences), consisted of a 2D-gantry with a jet printing unit. The printing process involved filling a metal cartridge with approximately 50 g of ink, pre-tempered to 33 ± 0.1 °C, and mounting it to a pre-heated aluminum jacket at the same temperature. The ink was conveyed pneumatically to a temperature-controlled micro-valve with a diameter of 300 *µ*m (SMLD 300 G Mikorventile Gyger, Fritz Gyger AG, Gwatt, Switzerland), set to 33.0 ± 0.1 °C. Bitmap images of dot patterns were rasterized in Labview software (Labview 2019, version 19.0.1f5), which calculated and configured valve opening time and coil currents. Movement commands were translated to the gantry system, where the bitmap was interpreted in a line-by-line fashion, and exact printer position was registered with an encoder signal and stored in the print controller. When the printer scanned a printable position, a trigger signal from the print controller forced the valve-controller to activate the magnetic force. Drop formation was obtained by the “peak-and-hold” procedure, whereby the micro valve was actuated electromagnetically with an opening current for an opening time, and the valve ball was pulled backwards to let the medium emerge at a holding current for a defined time that determines drop formation (see Fig. 7c). To speed up the printing process, the direction of the movement was inverted after every printed line. To ensure exact positioning of the dots, drop deflections due to different times of flight, flight directions or printable volume were corrected in the Labview software to guarantee shape fidelity and to impede drop deformation (e.g., satellite drops). All current and opening/holding times were adjusted to intricately control drop volume (390 ± 10 µg) and drop detachment. Piston height was kept constant at 2 cm, lateral motion was set at 50 mm/s in x-direction and at 200 mm/s for the y-axis movement. A diagram of this process is given in Fig. 7 and a summary of the print settings can be extracted from Supplementary Table 1.

**Fig. 8:**
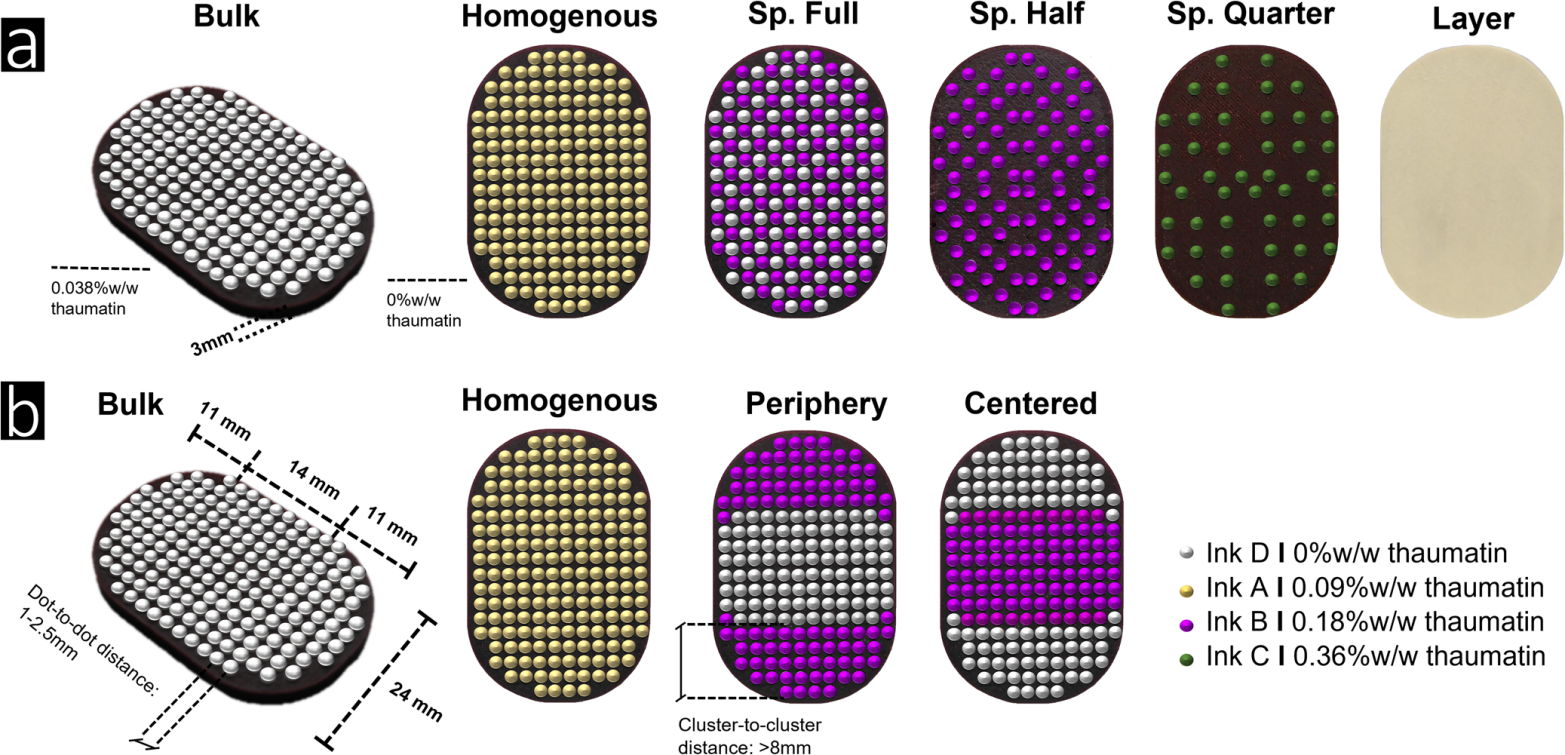
Experimental sample designs. Overview of **a** the six samples used to study the impact of micro-structuring on sweetness intensity and **b** the four samples used to study the impact of mesoscale level clustering on perception. Based on dot-to-dot distance, samples were either classified as micro- (*<* 2.5 mm) or mesoscale (*>* 8 mm cluster-to-cluster distance). Samples *Bulk*, *Homogeneous,* and *Spaced Full (Sp. Full)* were visually indistinguishable, varying in composition and dot pattern (ink A). Samples *Sp. Half* and, *Sp. Quarter* varied in total number of dots and, therefore had a different appearance. Differences in number of dots were compensated using ink with a higher concentration, ink B for *Sp. Half* and ink C for *Sp. Quarter*, respectively. The sample *Layer* was not jetted, but covered with a defined layer of ink A. In addition to *Homogeneous* and *Bulk*, samples *Periphery* and *Centered* were introduced to study the impact of mesoscale level structuring pooling thaumatin either at the center or at the periphery of the sample. The geometric design of all samples is shown in **b**.

Six samples were designed for sensory evaluation, investigating the effect of varying microscopic dot patterns (Fig. 8a). Micro-structured patterns had a maximum dot-to-dot distance of 2.5 mm. While the quantity of thaumatin remained constant, its placement varied between surface and bulk. Samples *Bulk*, *Homogeneous* and *Spaced Full* comprised 184 dots, sample *Spaced Half* 92 dots and sample *Spaced Quarter* 46 dots. Thaumatin concentration was 0 %w/w in sample *Bulk* (ink D), 0.09%w/w (ink A) in sample *Homogeneous*, 0.18%w/w in both samples *Spaced Full* and *Spaced Half*, and 0.36%w/w in sample *Spaced Quarter*. Sample *Layered* was not printed; instead, it was manually coated with a thin layer of ink A to obtain a consistent sweetness load.

In addition, four samples were designed to study the impact of meso- structured patterns on sweetness intensity (Fig. 8b). Overall thaumatin load was kept constant, while thaumatin was either distributed in the bulk or located in dots. Two more printing patterns, *Centered* and *Periphery*, were designed, each containing 184 dots. A gradient of sweetness from the center to the edge was established in both samples *Centered* and *Periphery* using ink B and D. These samples were categorized as meso-structured as the gap between contrasting dots exceeded 8 mm. Before sensory evaluation, all samples were stored for a maximum of seven days in the fridge (4 °C).

### Tongue surface reconstruction and emulation of dot

#### Melting

In order to examine the process of dot melting during licking, a tongue-emulating elastomeric surface was created. Surfaces were reconstructed from acquired human tongue images. For each subject, an average of ten images were acquired at the Decision Neuroscience Laboratory of the ETH Zurich. These images were collected from 50 participants who were undergoing an elec- troencephalography test (ethically approved with BASEC no. 2021-02420). To enhance contrast, each tongue was stained with a brilliant food dye solution based on the Denver papillae protocol^42^. Papillae segmentation and separation (see Supplementary Fig. 2) was accomplished with an automatic MATLAB code (MATLAB, MathWorks, version R2021a). This process converted the tongue into three regions of interest: filiform papillae, fungiform papillae, and the tongue surface. Following the Denver papillae protocol, fungi-form papillae were counted manually using Image J (version 1.54b). Unlike fungiform density per area derived from manual counting, single filiform papillae could not be distinguished. Therefore, a filiform number density was theoretically calculated dividing area density by both filiform radius r_Fi_ (180 ± 20 µm), extracted from Andablo-Reyes et al.^43^, and minimum papillae- to-papillae distance. In addition to number densities obtained from image analysis, heights of fungiform h_Fu_ (390 ± 72 µm) and filiform h_Fi_ (195 ± 6 µm) papillae were extracted from Andablo-Reyes et al.^43^. These values were employed to construct a biomimetic tongue surface, emulating the real surface. Papillae were randomly distributed, and their 3D shape was approximated to papillae forms, according to the following equation:

1 $$z=\,\frac{{x}^{2}}{{r}^{2}}+\frac{{y}^{2}}{{r}^{2}}$$,

where a structured 3D grid was created based on the x, y, and z values. The final object file was imported and converted in the Blender software (version 3.4.1). This led to the generation of a negatively extruded surface, which was subsequently printed using a SL Printer (Original Prusa SL1S SPEED 3D, Prusa Research, Czech). Prior to casting, the hard resin was treated with polyvinyl alcohol for 30 min at 80 °C. Following this, the PDMS Sylgard 184 Silicone cross-linking agents were mixed in a 10:1 % w/w ratio, cast onto the hard resin, and cured for three hours at 70 °C. A visualized workflow can be obtained from Supplementary Fig. 2.

To emulate saliva, PBS Buffer and water mixtures were used. Sample consumption was replicated by shearing the chocolate sample with a modified 3D printer (model MH300R1, ORD solutions, Canada) at a velocity v_T_ of 10 mm/s, under a normal force F_N_ of 1 N, and with a sample-to-tongue amplitude A/2 of 5 mm (see Supplementary Fig. 3). To regulate the temperature, the elastomer was heated, while dot melting was tracked using a high-speed camera, placed perpendicular to the motion of the sample.

#### Sensory evaluation

Fourteen participants (seven women and seven men aged between 23 and 36) took part to study the impact of microscale level variations on sweetness. The second part, which focused at discerning the influence of dot patterns at mesocale level on sweetness intensity, was conducted with seventeen participants (nine women and eight men, all aged between 23 and 36). Prior to being enrolled in the study, all participants gave their written consent, affirming that they understood the objective, the confidentiality measures concerning their data, and any risks associated with their participation. They were provided the opportunity to pose any questions, or to withdraw from the study without needing to elaborate on their reasons. All participants underwent preliminary training to evaluate their ability to identify sweetness. They attended two training sessions focused on familiarizing them with a unipolar scale, four training sessions for the first part of the study and three training sessions for the second part. During scale training, participants familiarized themselves with sweetness, testing a set of samples with defined sweetness intensities (anchor points). Training and test sessions took place within three weeks. During these tests, participants rated sample intensity at three consecutive time points (t_start_, t_max_ and t_end_) on a scale for sensation strength from 0 (not sweet) to 100 (extremely sweet). Participants were asked to place the samples longitudinally on the anterior half of the tongue with the dots/layer facing downwards, as shown in Fig. 5a–c. The sensation was initiated by gently pressing the sample to the palate without biting it. The tongue was moved periodically to enhance the melting sensation. At the end of the sensation and if required during testing, swallowing was allowed. After each sample, the mouth was rinsed with lukewarm water and neutralized with a cracker (M-Classic Microc, Migros SA, Switzerland).

Each session started with a warm-up sample of defined maximum sweetness intensity I_max_ of 50, and samples were presented in balanced order to exclude sequence effects. During test sessions, participants were asked to start their evaluation after putting the sample in their mouth, and to note I_max_, i.e., the maximum perceived sweetness intensity over the course of testing. In addition to I_max_, participants were instructed to rate initial (I_start_) and final (I_end_) sweetness sensation on a scale bar between 0 (not sweet) and 100 (extremely sweet). Initial sweetness intensity was defined as the first perception of sweetness, once the dots had started to melt. Final sweetness intensity I_end_ was defined as the last sweetness sensation before the last piece of the sample had been completely swallowed. Due to COVID pandemic, both training and testing sessions were carried out at home. To ensure uniform conditions, subjects repeated the sessions under similar conditions regarding time of day, place of consumption, sample preparation, and room temperature. To facilitate consumer guidance and guarantee data recording, participants were guided through the sessions using EyeQuestion (licensed to BFH-HAFL, Zollikofen, Switzerland). At the end of each session, an individualized feedback was provided, and after the third training session, a personalized feedback was generated and discussed with every participant via online video conferencing.

### Data analysis

Data was collected with EyeQuestion software (Logic8 B.V., NK Elst, Netherlands). Two-way ANOVA was performed with EyeOpen R, where sweetness intensity was set as dependent variable, samples and time-independent points (I_start_, I_max_, I_end_) were treated as fixed factors, while assessors and replicates were treated as random factors. For significant results (*p* < 0.05), a two-sided comparison with a post-hoc Tukey test was performed.

### Reporting summary

Further information on research design is available in the Nature Research Reporting Summary linked to this article.

### Supplementary information

Supplementary Information

Reporting Summary

## Data Availability

The authors declare that all pertinent data that support this study have been included within the paper. Raw data will be made available by the corresponding author upon request. The code used in this research can be obtained from github.com/burkardj/npj Conversion.git.
