# Supplementary material for: Inkjet-based surface structuring: amplifying sweetness perception through additive manufacturing in foods
Source: NPJ Sci Food. 2023 Aug 18;7:42. doi: 10.1038/s41538-023-00218-x (PMC10439107; doi:10.1038/s41538-023-00218-x)
Supplement: Supplementary file 1 — Supplementary Information [file 41538_2023_218_MOESM1_ESM.pdf]

# Supplementary Information:
# Inkjet-based Surface Structuring: Amplifying Sweetness Perception through Additive Manufacturing in Foods

Johannes Burkard[1,2*], Lucas Kohler[1], Tanja Berger[1], Mitsuko Logean[1], Kim Mishra[1], Erich J. Windhab[1] and Christoph Denkel[2]

[1]Insitute of Food, Nutrition and Health, ETH Zürich, Schmelzbergstrasse 9, 8092 Zürich, Switzerland.
[2]School of Agricultural, Forest and Food Sciences, Food Science and Management, Bern University of Applied Sciences, Länggasse 85, 3052 Zollikofen, Switzerland.

*Corresponding author(s). E-mail(s): johannes.burkard@hest.ethz.ch;

## Contents

**Supplementary Table 1** **2**

**Supplementary Note 1** **3**

**Supplementary Figure 1** **4**

**Supplementary Figure 2** **5**

**Supplementary Figure 3** **6**

# Supplementary Table 1

| Print Settings | |
|---|---|
| **Movement Control** | |
| Velocity X-Axis (mm/s) | 30 |
| Velocity Y-Axis (mm/s) | 50 |
| Acceleration X-Axis ($mm/s^2$) | 1000 |
| Acceleration Y-Axis ($mm/s^2$) | 1000 |
| **Valve Control** | |
| Peak time $t_P$ (µs) | 400 |
| Holding time $t_H$ (µs) | 1100 |
| Peak current $I_P$ (mA) | 700 |
| Holding current $I_H$ (mA) | 237 |

**Supplementary Table 1**: Print settings of gantry printer. Each dot pattern was designed as a bitmap, where white pixels represented printable dots and black pixels empty spaces. The bitmap images were rasterized in Labview software (Labview 2019, version 19.0.1f5), where both opening time of the valve and coil currents within the valve controller were calculated and configured (see Figure 7 A - D).

# Supplementary Note 1

**Dot Contour Plotting** The dot shape for inks A to D was visually inspected using a digital camera (Nikon D5300, Nikon, Japan). This evaluation involved assessing dot shape from a top view, and dot height and form from a side view. The captured images were subsequently processed using Jupyter Notebook. In the initial stages, images underwent pre-processing with a Gaussian filter before being cropped. Subsequently, contours were identified using the *find contours* command. These contour coordinates were then fitted to a second-degree polynomial function with the help of the *polyval* command. An average of ten points was collected per ink/pattern to construct a definitive polynomial function. By confirming the circular base shape of the dots and integrating it with the polynomial fit, the dots' surface was reconstructed and visualized. Stimulus size ($A_{pattern}$) was calibrated according to the adjusted dot area. A comprehensive overview of samples created using different inks and patterns is displayed in Supplementary Figure 1. The code can be obtained from github.com/burkardj/npj_Conversion.git.

# Supplementary Figure 1

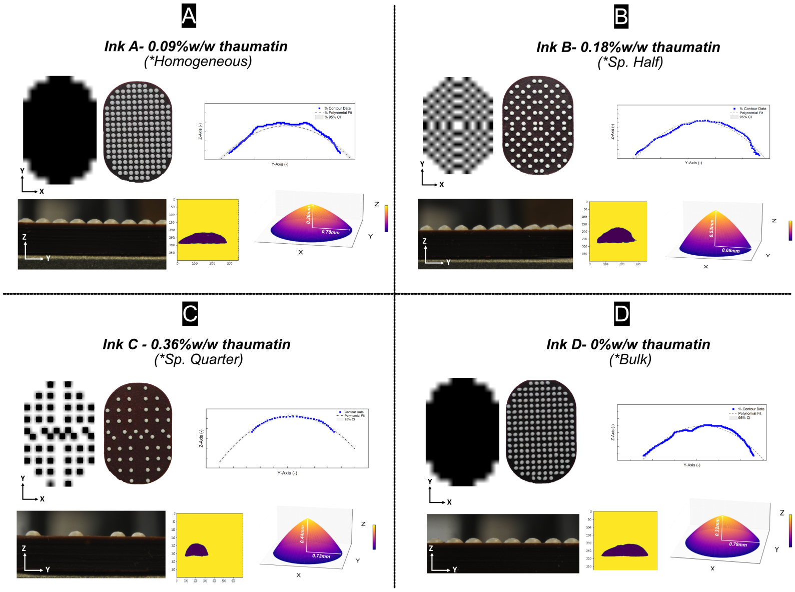


**Supplementary Figure 1**: Side and top views of samples *Homogeneous* (A), *Sp. Half* (B), *Sp. Quarter* (C) and *Bulk* (D). These samples were printed with dots of ink compositions ranging from 0 (*Bulk*) to 0.36 % w/w (*Sp. Quarter*). For each pattern, both the bitmap and the top view image of the dot pattern are displayed. Side view images were processed using a Gaussian filter for binarization and cropping. Dot contours were analyzed and fitted with a polynomial equation. The dots were then surface reconstructed by averaging the dot diameter from the top view images and using the fitted polynomial contour equation, with the color band indicating the z-position.

# Supplementary Figure 2

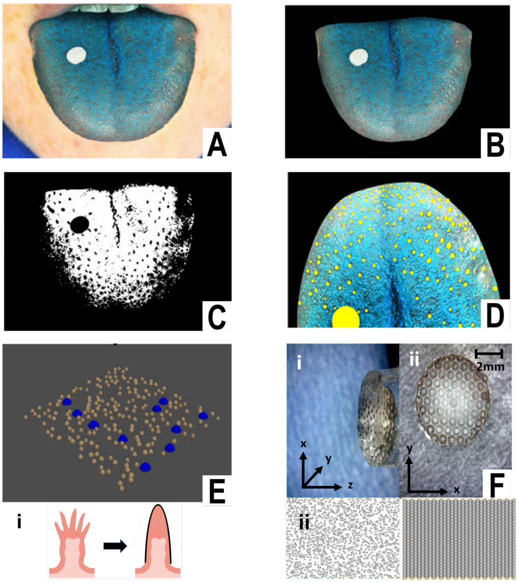


**Supplementary Figure 2**: Workflow steps: (A) Tongue image acquisition with white paper circles used a scale reference. (B-C) Pre-processing of tongue images with MATLAB to separate the tongue into three regions, i.e. the filiform papillae, the tongue surface and the fungiform papillae. (D) The yellow colored fungiform papillae were counted manually according to the Denver papillae protocol. (E) Randomized surfaces were generated based on papillae densities and a geometric design approximation. Due to resolution limitations, filiform papillae (i) with protruding hairs were approximated with a paraboloid shape. (F) Casted elastomers mimicking the tongue surface at two separate perspectives and for two different surface densities. (i) Representative tongue elastomer with densities similar to the tongue tip. (ii) Tongue elastomer with a isotropic distribution of fungiform papillae.

# Supplementary Figure 3

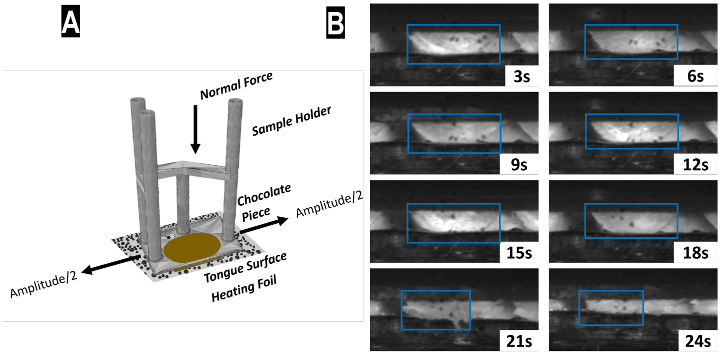


**Supplementary Figure 3**: A) Scheme of the in-vitro chocolate melting setup. The dotted chocolate pieces were mounted on a 3D printed holder attached to a modified 3D printer (ORD solutions, model MH300R1). Sample consumption was mimicked by shearing the chocolate sample at a velocity $v_T$ of 10 mm/s, at a normal force $F_N$ of 1 N, and over a sample-to-tongue amplitude A/2 of 5 mm. Human tongue movement and in-mouth normal force have been estimated to be in the range used in our experimental setup [1–3]. B) Dot melting at different stages during in-vitro oral consumption is visualized with reference to the blue-framed dot and was monitored with a high speed camera, set perpendicular to the sample movement. A set of selected images from a time grid at different stages of shearing, from 0 s (no melting) to 24 s (complete melting) are shown.

# References

[1] Laiho S, Williams RPW, Poelman A, Appelqvist I, Logan A. Effect of whey protein phase volume on the tribology, rheology and sensory properties of fat-free stirred yoghurts. Food Hydrocolloids. 2017;67:166–177. https://doi.org/10.1016/j.foodhyd.2017.01.017.

[2] Prinz JF, de Wijk RA, Huntjens L. Load dependency of the coefficient of friction of oral mucosa. Food Hydrocolloids. 2007;21(3):402–408. https://doi.org/10.1016/j.foodhyd.2006.05.005.

[3] Miller JL, Watkin KL. The influence of bolus volume and viscosity on anterior lingual force during the oral stage of swallowing. Dysphagia. 1996;11(2):117–124. https://doi.org/10.1007/BF00417901.
