# Supplementary material for: Inkjet-based surface structuring: amplifying sweetness perception through additive manufacturing in foods
Source: NPJ Sci Food. 2023 Aug 18;7:42. doi: 10.1038/s41538-023-00218-x (PMC10439107; doi:10.1038/s41538-023-00218-x)
Supplement: Supplementary file 2 — Reporting Summary [file 41538_2023_218_MOESM2_ESM.pdf]

nature portfolio

Corresponding author(s): Johannes Burkard

Last updated by author(s): Jul 28, 2023

Reporting Summary

Nature Portfolio wishes to improve the reproducibility of the work that we publish. This form provides structure for consistency and transparency in reporting. For further information on Nature Portfolio policies, see our Editorial Policies and the Editorial Policy Checklist.

Statistics

For all statistical analyses, confirm that the following items are present in the figure legend, table legend, main text, or Methods section.

n/a	Confirmed	
☐	☒	The exact sample size (n) for each experimental group/condition, given as a discrete number and unit of measurement
☐	☒	A statement on whether measurements were taken from distinct samples or whether the same sample was measured repeatedly
☐	☒	The statistical test(s) used AND whether they are one- or two-sided<br>*Only common tests should be described solely by name; describe more complex techniques in the Methods section.*
☐	☒	A description of all covariates tested
☐	☒	A description of any assumptions or corrections, such as tests of normality and adjustment for multiple comparisons
☐	☒	A full description of the statistical parameters including central tendency (e.g. means) or other basic estimates (e.g. regression coefficient) AND variation (e.g. standard deviation) or associated estimates of uncertainty (e.g. confidence intervals)
☐	☒	For null hypothesis testing, the test statistic (e.g. F, t, r) with confidence intervals, effect sizes, degrees of freedom and P value noted<br>*Give P values as exact values whenever suitable.*
☒	☐	For Bayesian analysis, information on the choice of priors and Markov chain Monte Carlo settings
☒	☐	For hierarchical and complex designs, identification of the appropriate level for tests and full reporting of outcomes
☐	☒	Estimates of effect sizes (e.g. Cohen's d, Pearson's r), indicating how they were calculated

Our web collection on statistics for biologists contains articles on many of the points above.

Software and code

Policy information about availability of computer code

Data collection	git@github.com:burkardj/npj_Conversion.git
Data analysis	git@github.com:burkardj/npj_Conversion.git

For manuscripts utilizing custom algorithms or software that are central to the research but not yet described in published literature, software must be made available to editors and reviewers. We strongly encourage code deposition in a community repository (e.g. GitHub). See the Nature Portfolio guidelines for submitting code & software for further information.

Data

Policy information about availability of data

All manuscripts must include a data availability statement. This statement should provide the following information, where applicable:

- Accession codes, unique identifiers, or web links for publicly available datasets
- A description of any restrictions on data availability
- For clinical datasets or third party data, please ensure that the statement adheres to our policy

Included in Chapter 05

Research involving human participants, their data, or biological material

Policy information about studies with human participants or human data. See also policy information about sex, gender (identity/presentation), and sexual orientation and race, ethnicity and racism.

Reporting on sex and gender	Fourteen participants (seven women and seven men aged between 23 and 36) took part to study the impact of microscale level variations on sweetness. The second part, which focused at discerning the influence of dot patterns at mesocale level on sweetness intensity, was conducted with seventeen participants (nine women and eight men, all aged between 23 and 36)
Reporting on race, ethnicity, or other socially relevant groupings	No socially relevant groupings in our sensory study
Population characteristics	See above
Recruitment	Recruited from ETH platform and via colleagues
Ethics oversight	ETH Zurich / Bern University of Applied Sciences

Note that full information on the approval of the study protocol must also be provided in the manuscript.

Field-specific reporting

Please select the one below that is the best fit for your research. If you are not sure, read the appropriate sections before making your selection.

☐ Life sciences ☒ Behavioural & social sciences ☐ Ecological, evolutionary & environmental sciences

For a reference copy of the document with all sections, see nature.com/documents/nr-reporting-summary-flat.pdf

Behavioural & social sciences study design

All studies must disclose on these points even when the disclosure is negative.

Study description	Impact of varying tastants, i.e. thaumatin a model sweetener, with AM. Data were generated with a sensory panel of 14 to 17 people, progressively profiling sweetness intensity
Research sample	ETH Zurich students, citizens of Switzerland, age 23-36, nine women/eight men, representative group of people
Sampling strategy	Sample size was not pre-determined, but a minimum > 10 was selected to quantify a minimum of 60 responses per subject/item (20 for each time point).
Data collection	Data was recorded with EyeQuestion software (licensed by Bern University of Applied Sciences)
Timing	Start: Study 1 - 18.05.2021-30.05.2021 - End: 021.07.2021-2.08.2021
Data exclusions	No data were excluded
Non-participation	Nobody dropped out
Randomization	Allocation was not random, but sampling was balanced

Reporting for specific materials, systems and methods

We require information from authors about some types of materials, experimental systems and methods used in many studies. Here, indicate whether each material, system or method listed is relevant to your study. If you are not sure if a list item applies to your research, read the appropriate section before selecting a response.

Materials & experimental systems

n/a	Involved in the study
☒	☐ Antibodies
☒	☐ Eukaryotic cell lines
☒	☐ Palaeontology and archaeology
☒	☐ Animals and other organisms
☒	☐ Clinical data
☒	☐ Dual use research of concern
☒	☐ Plants

Methods

n/a	Involved in the study
☒	☐ ChIP-seq
☒	☐ Flow cytometry
☒	☐ MRI-based neuroimaging
